# Metabolism: getting things right!

**DOI:** 10.1038/s44318-024-00209-y

**Published:** 2024-09-16

**Authors:** William Teale, Daniel Klimmeck

**Affiliations:** EMBO, Heidelberg, Germany

**Keywords:** Metabolism, Methods & Resources

## Abstract

A new series of methods commentaries reflects on best practice in metabolomics research and how to support standards and productivity in the field.

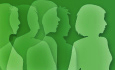

It is boomtime for metabolism and metabolomics research. One hundred years after Otto Warburg put metabolism at the core of his work on tumours, the analysis of metabolites has matured impressively and re-entered centre stage in diverse fields such as epigenetics, extracellular matrix tissue biology and development. This renewed interest has led to an unprecedented surge in laboratories using metabolomics to answer essential scientific questions, and myriad related studies pursued.

However, there are signs that this recent increase in attention is causing its own problems: the sharp up-tick in submitted metabolism manuscripts is stressing the system, as scientific journals now struggle to find enough referees with the appropriate expertise. As a result, senior researchers in the field are expressing major concerns, noting that current manuscripts and publications in metabolomics often lack the experimental design, data presentation and accurate interpretation required to meet long-established standards. These failings inevitably lead to inaccuracies that affect the credibility and usefulness of research.

These issues prompted the editorial team at The EMBO Journal to launch a series of methods commentaries on metabolomics and metabolism research, with the aim of providing informed viewpoints and guidance from respected voices on how to pursue plausible, reproducible and original experimentation.

The first piece by Meiser and Frezza (2024) highlights frequent pitfalls of metabolism data presentation and offers practical suggestions for designing, processing, and communicating metabolomics analyses. Next, David James and team reflect on the astounding impact mouse genetics can have on the outcome of metabolic studies, demonstrating how the use of animals with diverse genetic backgrounds helps reveal novel mechanisms and dissect phenotypes of interest (Masson et al, 2024). Orian Shirihai and colleagues revisit the concepts of fuel preference in cell metabolism and explore appropriate experimental strategies to assess it. An overview of critical steps and considerations for setting up and running in vivo metabolite tracing studies in the clinic is provided by Faubert and Tasdogan. Additional perspectives in the series will focus on best practices when recapitulating authentic tissue metabolite conditions in cell culture, optimizing hypoxia assays, comparing ex vivo vs in situ 3D metabolomics, and exploring the synthesis of high-value plant secondary metabolites.

We hope this commentary series will inspire newcomers and established scientists alike to explore and describe this exciting yet technically challenging area of research. Most of all, we hope that it will support high standards and productive exchange in the field. Everyone interested in metabolism should benefit from discussions on ‘how to get it right’!

## References

[CR1] Masson SWC, Cutler HB, James DE (2024) Unlocking metabolic insights with mouse genetic diversity. EMBO J. 10.1038/s44318-024-00221-2 10.1038/s44318-024-00221-2 PMC11535531 39284908

[CR2] Meiser J, Frezza C (2024) Presenting metabolomics analyses: what’s in a number? EMBO J. 10.1038/s44318-024-00098-1 10.1038/s44318-024-00098-1 PMC11480362 38664540

